# Sulfonic Acid Functionalized Nano-**γ**-Al_**2**_O_**3**_: A New, Efficient, and Reusable Catalyst for Synthesis of 3-Substituted-2*H*-1,4-Benzothiazines

**DOI:** 10.1155/2013/838374

**Published:** 2013-07-14

**Authors:** Wei Lin Li, Shuan Bao Tian, Feng Zhu

**Affiliations:** ^1^Department of Medicinal Chemistry, Pharmacy College of Xinxiang Medical University, Xinxiang 453003, China; ^2^School of Basic Medical Sciences, Xinxiang Medical University, Xinxiang 453003, China; ^3^Department of Urology, The First Affiliated Hospital of Xinxiang Medical University, Xinxiang 453003, China

## Abstract

A simple and efficient synthetic protocol has been developed for the synthesis of 3-substituted-2*H*-1,4-benzothiazines by using a novel sulfonic acid functionalized nano-**γ**-Al_2_O_3_ catalyst, devoid of corrosive acidic, and basic reagents. The developed method has the advantages of good to excellent yields, short reaction times, operational simplicity, and a recyclable catalyst. The catalyst can be prepared by a simple procedure from inexpensive and readily available nano-**γ**-Al_2_O_3_ and has been shown to be recoverable and reusable up to six cycles without any loss of activity.

## 1. Introduction

1,4-Benzothiazine derivatives are important biologically and pharmaceutically active heterocyclic compounds. They have received considerable attention in the field of pharmaceutical industry owing to their broad range of biological activities such as antifungal [1, 2], immunostimulating [3], antirheumatic [4], antiallergic [5], and antitumor activities [6]. 1,4-Benzothiazines are also active on the cardiovascular system, and the vasorelaxant, antiarrhythmic, and antihypertensive effects have been reported [7–11]. 1,4-Benzothiazines induced neurotoxic or neuroprotective effects have been described and a possible role in neurodegenerative diseases has been hypothesized [12, 13]. 1,4-Benzothiazines provide privileged scaffolds in lead identification/drug discovery programs and have provided therapeutically useful compounds in fields such as anti-rheumatic agents (e.g., MX-68 [4]), histamine H_1_-receptor antagonists (e.g., VUF-K-8788 [5]), aldose reductase inhibitors, which are very promising for treating hyperglycemia (e.g., SPR-210 [14]), and Ca^2+^ channel antagonists (e.g., semotiadil fumarate [11]).

There are several methods available for the preparation of 1,4-benzothiazine derivatives including the ring expansion of benzothiazoles or benzothiazolines [15, 16], basic mediated reactions of *o*-aminothiophenols with *ω*-bromoacetophenones [17], HCl-catalyzed reactions of *o*-nitrobenzenesulfenyl chlorides with ketones [18], treatment of aminothioalkenols with *p*-TsOH or H_3_PO_4_ [19], reaction of bis(*o*-aminophenyl)-disulfide with ketones [20], the condensation of *o*-aminothiophenols and 2-bromo-1-aryl-ethanones using KHSO_4_ [21], and simultaneous reduction of nitro group and S–S bond in nitrodisulfides induced by low-valent titanium reagent [22]. Despite the importance of these reported protocols many suffer from drawbacks such as the use of expensive reagents, harsh reaction conditions, prolonged reaction times, cumbersome product isolation procedures, low yields more stoichiometric amount of catalyst. Hence, to explore a mild, efficient, and environmentally benign recyclable synthetic protocol for the 1,4-benzothiazine derivatives is highly desirable.

In recent times, the development of environmentally benign, green, and easily recyclable catalyst for the production of fine chemicals has been an area of growing interest. In this context, solid acid catalysts play prominent role in organic synthesis under heterogeneous reaction conditions. Various solid acid catalysts like zeolite [23], heteropoly acids [24], Amberlyst-15 [25], Nafion-H [26], silica sulfuric acid [27], silica phosphoric acid [28], and silica supported perchloric acid [29] with lower toxicity, high stability, and recyclability have attracted more attention. 

**Scheme 1:**
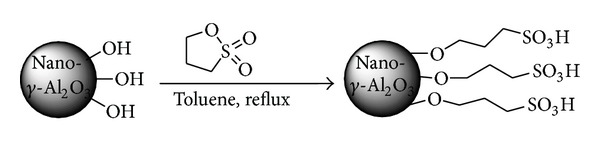
Synthesis of sulfonic acid functionalized nano-*γ*-Al_2_O_3_.

As a part of our endeavors towards the development of efficient and environmentally benign synthetic methodologies using economic and eco-friendly heterogeneous catalysts [27, 29], we have investigated the synthesis of 3-substituted-2*H*-1,4-benzothiazines from *o*-aminothiophenols and *ω*-bromoketones in the presence of a novel sulfonic acid functionalized nano-*γ*-Al_2_O_3_ catalyst in EtOH at reflux temperature (Scheme 1).

## 2. Results and Discussion

**Scheme 2:**
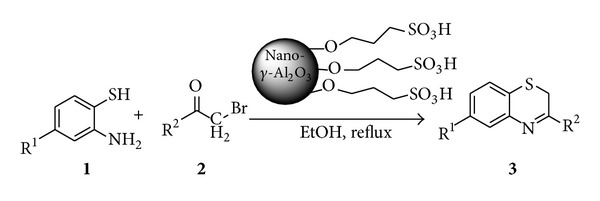
Synthesis of 3-substituted-2*H*-1,4-benzothiazines using sulfonic acid functionalized nano-*γ*-Al_2_O_3_.

Sulfonic acid functionalized nano-*γ*-Al_2_O_3_ was easily prepared by the reaction of nano-*γ*-Al_2_O_3_ with 1,3-propanesultone (Scheme 2), and it was characterized by FT-IR, X-ray powder diffraction (XRD), thermogravimetric analysis (TGA), and transmission electron microscopy (TEM). The amount of sulfonic acid loaded on the surface of nano-*γ*-Al_2_O_3_ is determined by TG analysis and confirmed by ion-exchange pH analysis.

**Figure 1:**
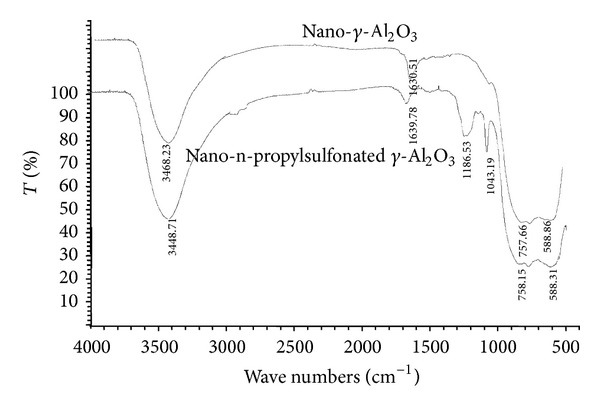
FTIR spectra of nano-*γ*-Al_2_O_3_ (up) and sulfonic acid functionalized nano-*γ*-Al_2_O_3_ (down).

Figure 1 presents the FT-IR spectra of nano-*γ*-Al_2_O_3_ and sulfonic acid functionalized nano-*γ*-Al_2_O_3_. As shown in this figure, the presence of an extra sulfonic acid group in the sulfonic acid functionalized nano-*γ*-Al_2_O_3_ increases the number of vibrational modes and brought completely different FT-IR spectrum. The FT-IR spectra of sulfonic acid functionalized nano-*γ*-Al_2_O_3_ exhibit two characteristic peaks at 589 cm^−1^ and 758 cm^−1^ due to the stretching vibrations of Al–O bond in *γ*-Al_2_O_3._ Moreover, two important peaks at 1043 cm^−1^ and 1187 cm^−1^ are assigned to S–O stretching vibration. The broad peak at 3444 cm^−1^ belongs to the stretching of OH groups in the SO_3_H. These results indicate that the reaction of nano-*γ*-Al_2_O_3_ with 1,3-propanesultone succeeds in incorporating sulfated groups in nano-*γ*-Al_2_O_3_.

**Figure 2:**
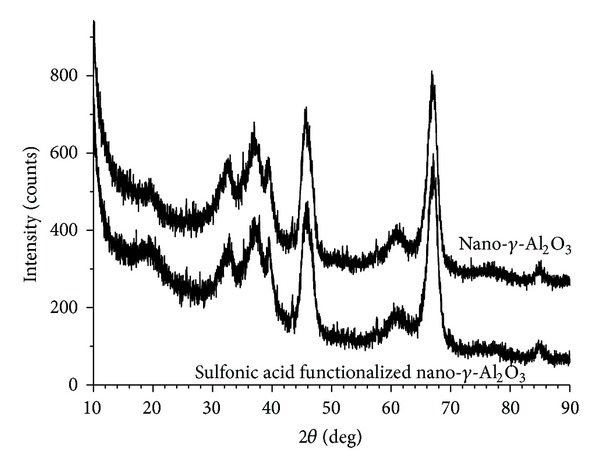
XRD patterns of nano-*γ*-Al_2_O_3_ (up) and sulfonic acid functionalized nano-*γ*-Al_2_O_3_ (down).

XRD measurements of nano-*γ*-Al_2_O_3_ and sulfonic acid functionalized nano-*γ*-Al_2_O_3_ exhibit diffraction peaks at around 19.5, 32.6, 36.6, 39.5, 45.8, 60.6, and 67.2 corresponding to the (111), (220), (311), (222), (400), (511), and (440) faces (Figure 2). The observed diffraction peaks agree well with the cubic structure of *γ*-Al_2_O_3_ (JCPDS file number 29-0063). It is clear that the ordered structure of nano-*γ*-Al_2_O_3_ is retained after introducing the propylsulfonic acid group. The average crystallite sizes are calculated to be 14.9 nm using the Scherrer equation, which are in good accordance with TEM results.

**Figure 3:**
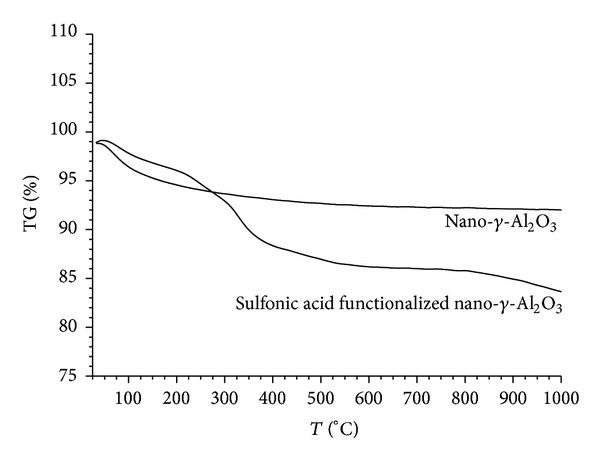
TG analysis of nano-*γ*-Al_2_O_3_ (up) and sulfonic acid functionalized nano-*γ*-Al_2_O_3_ (down).

The stability of the nano-*γ*-Al_2_O_3_ and sulfonic acid functionalized nano-*γ*-Al_2_O_3_ is determined by thermogravimetric analysis (Figure 3). A significant decrease in the weight percentage of the nano-*γ*-Al_2_O_3_ and sulfonic acid functionalized nano-*γ*-Al_2_O_3_ at about 150°C is related to desorption of water molecules from the catalysts surface. In the TG curve of sulfonic acid functionalized nano-*γ*-Al_2_O_3_, complete loss of all the covalently attached organic structures is seen in the temperature range of 230–960°C. The shouldering observed from 328°C onwards may be due to the decomposition of alkyl-sulfonic acid groups. According to the TGA, the amount of sulfonic acid functionalized nano-*γ*-Al_2_O_3_ is evaluated to be 0.78 mmol*·*g^−1^. This result is in agreement with that of ion-exchange pH analysis.

**Figure 4:**
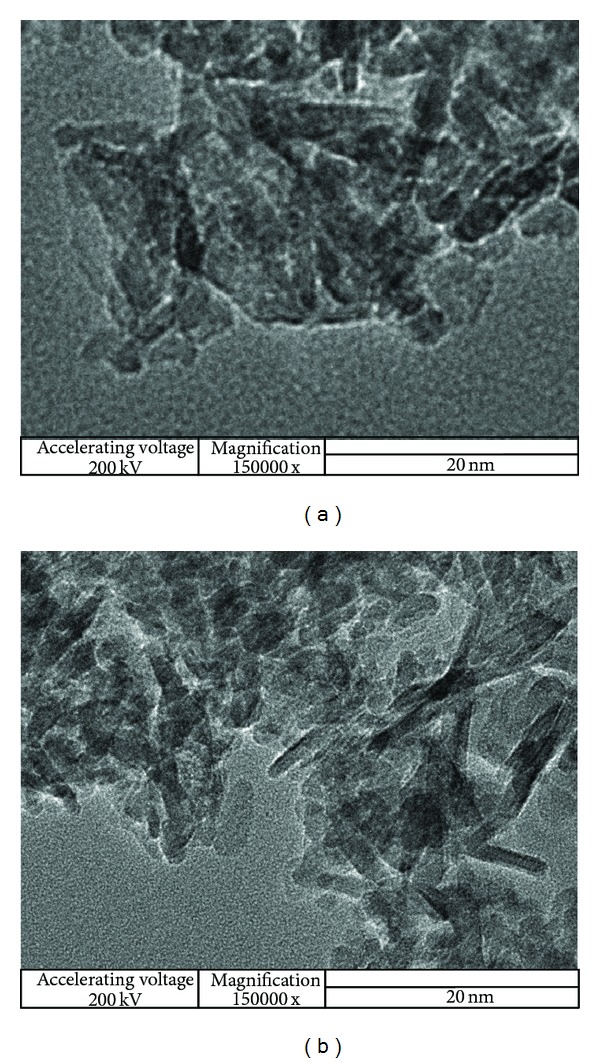
(a) TEM images of nano-*γ*-Al_2_O_3_. (b) TEM images of sulfonic acid functionalized nano-*γ*-Al_2_O_3_.

The sizes of nano-*γ*-Al_2_O_3_ and sulfonic acid functionalized nano-*γ*-Al_2_O_3_ are further analyzed by TEM and the results (Figures 4(a) and 4(b)) showed the nanoparticles have nanodimensions ranging from 10 to 20 nm. In TEM images, the shapes of *γ*-Al_2_O_3_ particles are relatively round, and those of treated n-propylsulfonated *γ*-Al_2_O_3_ are rather rectangular, which is attributed to the presence of sulfonic acid groups covalently attached to the *γ*-Al_2_O_3_ surfaces.

To achieve suitable conditions for the synthesis of 3-substituted-2*H*-1,4-benzothiazines, we tested the reaction of *o*-aminothiophenol **1** with 2-bromo-1-phenyl-ethanone **2** as a simple model system in EtOH at reflux temperature using various catalysts (Table 1). As could be seen in Table 1, the best result was obtained with 50 mg/mmol of sulfonic acid functionalized nano-*γ*-Al_2_O_3_ as the catalyst in EtOH at reflux temperature (entry 3). Using less catalyst resulted in lower yields, whereas higher amounts of catalyst did not affect reaction times and yields. When this reaction was carried out without sulfonic acid functionalized nano-*γ*-Al_2_O_3_ or nano-*γ*-Al_2_O_3,_ the yield of the expected product was low. In the presence of *p*-TsOH, sulfamic acid, or silica sulfuric acid, the product was obtained in moderate yield.

To find the optimal solvent for this reaction, the model reaction was carried out at reflux temperature using EtOH, H_2_O, CH_2_Cl_2_, THF, and CH_3_CN as solvents. It is shown in Table 2 that the reaction using EtOH (96%) or CH_3_CN (97%) as the solvents gave the corresponding product 3-phenyl-2*H*-1,4-benzothiazine in high yields (Table 2, entries 8 and 2). From the economic and environmental point of view, EtOH was chosen as the reaction medium for all further reactions. Furthermore, the relation between the yields of the model reaction and temperature was also studied. We carried out the reaction at temperatures ranging from 25°C to reflux temperature using EtOH as the reaction medium (Table 2, entries 5–8), finding that the yields of desired product 3-phenyl-2*H*-1,4-benzothiazine were improved as the temperature was increased. Therefore, the best reaction conditions were obtained in EtOH under refluxed temperature.

In order to demonstrate the versatility of the sulfonic acid functionalized nano-*γ*-Al_2_O_3_ promoted synthesis of 3-substituted-2*H*-1,4-benzothiazines, a series of *ω*-bromoketones were treated with various *o*-aminothiophenols (Table 3). The reactions proceeded in EtOH at reflux temperature within a short time to afford the products. The reaction of *o*-aminothiophenol with various *ω*-bromoketones resulted in high yields of 3-substituted-2*H*-1,4-benzothiazines. The structures of the products were established from their spectral properties (IR, ^1^H NMR, and elemental analysis) and also by comparison with the available literature data.

**Figure 5:**
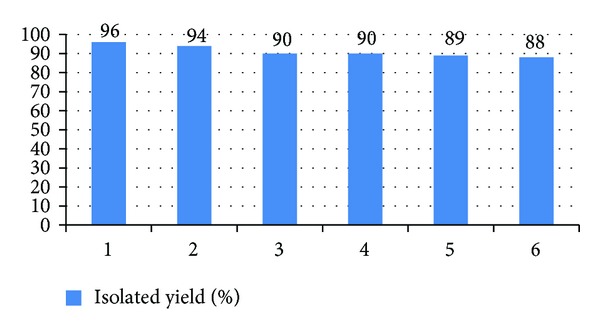
Reusability of sulfonic acid functionalized nano-*γ*-Al_2_O_3_ synthesis of 3-phenyl-2*H*-1,4-benzothiazine.

To demonstrate the recyclability of the catalyst, after each cycle the reaction mixture was allowed to cool and the catalyst was recovered by simple filtration, washed with EtOH, and dried in an oven at 100°C for 30 min prior to use. The catalyst was reused for the same reaction without further activation. The reaction proceeded smoothly even after six cycles, without any extension of reaction time or marked loss in yield (Figure 5).

**Scheme 3:**
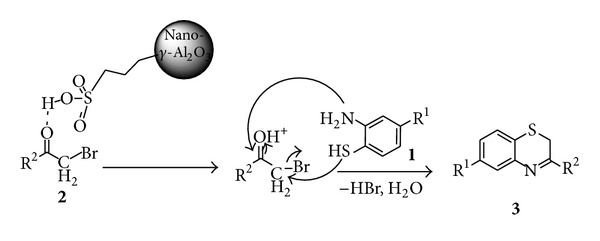
A plausible mechanism for the synthesis of 3-substituted-2*H*-1,4-benzothiazines.

The formation of product may be explained by the reaction of *ω*-bromoketone **2** with sulfonic acid functionalized nano-*γ*-Al_2_O_3_ which forms an oxonium ion. Later it reacts with *o*-aminothiophenol and subsequent cyclization result in expected product (Scheme 3).

## 3. Conclusion 

In conclusion, we have developed a novel and reusable sulfonic acid functionalized nano-*γ*-Al_2_O_3_ catalyst for an efficient synthesis of 3-substituted-2*H*-1,4-benzothiazines. The salient features of the present protocol are easy work-up, recyclability of the catalyst, and good yields. The present protocol offers a simple, inexpensive, and versatile approach to the synthesis of 3-substituted-2*H*-1,4-benzothiazines.

## 4. Experimental Part

### 4.1. Materials and Instrumentation

*γ*-Alumina powder with particle size at about 20 nm was purchased from Aladdin (Shanghai, China) and was used without further purification. Other reagents and starting materials were purchased from commercial resources and were used as received. All products were characterized by comparison of their spectral and physical data with those previously reported. Progress of the reactions was monitored by TLC.

XRD patterns were recorded using a Cu K*α* radiation source on a D8 Advance Bruker powder diffractometer. TEM studies were performed using a JEM 2100 transmission electron microscope on an accelerating voltage of 150 kV. TGA curves are recorded using a DT-40 thermoanalyzer. IR spectra were determined on FTS-40 infrared spectrometer. ^1^H NMR spectra were determined on Bruker AV-400 spectrometer at room temperature using tetramethylsilane (TMS) as an internal standard (CDCl_3_ solution); coupling constants (*J*) were measured in Hz. Elemental analysis was performed by a Vario-III elemental analyzer. Melting points were determined on an XT-4 binocular microscope and were uncorrected.

### 4.2. Synthesis of Sulfonic Acid Functionalized Nano-*γ*-Al_2_O_3_

Nano-*γ*-Al_2_O_3_ (6 g) was suspended in 600 mL of 0.1 M toluene solution of 1,3-propanesultone and the colloidal solution was refluxed for 48 h. The sulfonated nano-*γ*-Al_2_O_3_ was isolated and purified by repeated washing and centrifugation. It was characterized by FT-IR, XRD, TGA, SEM, and TEM. The amount of sulfonic acid loaded on the surface of nano-*γ*-Al_2_O_3_ was determined by TG analysis and confirmed by ion- exchange pH analysis.

### 4.3. Ion-Exchange pH Analysis

To an aqueous solution of NaCl (1 M, 25 mL) with a primary pH 5.93, the catalyst (500 mg) was added and the resulting mixture was stirred for 2 h after which the pH of solution decreased to 1.81. This is equal to a loading of 0.78 mmol SO_3_H*·*g^−1^.

### 4.4. General Procedure for the Synthesis of 3-Substituted-2*H*-1,4-Benzothiazines

To a suspension of a *ω*-bromoketones (1 mmol) and sulfonic acid functionalized nano-*γ*-Al_2_O_3_ (50 mg) in EtOH (10 mL), *o*-aminothiophenol (1 mmol) was added slowly and the mixture was stirred at reflux temperature. The reaction was monitored by TLC. After completion, the reaction mixture was filtered. The catalyst was washed with EtOH, dried, and reused for a consecutive run under the same reaction conditions. Evaporation of the solvent followed by recrystallization from EtOAc gave the desired product in good to high yields. 

### 4.5. Selected Spectral Data

*3-Phenyl-2H-1,4-benzothiazine* (**3a**). Mp: 46–48°C; IR (KBr) **ν**: 2928, 1638, 1463, 776 cm^−1^; ^1^H NMR (400 MHz, CDCl_3_): 7.46–6.90 (m, 9H, Ar), 3.82 (s, 2H, CH_2_); anal. calcd. for C_14_H_11_NS: C 74.63, H 4.92, N 6.22, S 14.23; found: C 75.02, H 4.99, N 6.19, S 14.20.

*3*-*(2*′*-Benzofuryl)-2H-1,4-benzothiazine* (**3k**). Mp: 85-86°C; IR (KBr) **ν**: 2933, 1669, 1472, 1246, 762 cm^−1^; ^1^H NMR (400 MHz, CDCl_3_): 8.02–6.91 (m, 7H, Ar), 3.67 (s, 2H, CH_2_); anal. calcd for C_12_H_9_NOS: C 66.95, H 4.21, N 6.51, S 14.90; found: C 67.02, H 4.18, N 6.54, S 14.97.

*6-Chloro-3-methyl-2H-1,4-benzothiazine* (**3l**). Oil; IR (KBr) **ν**: 2986, 2922, 1655, 1472, 1369, 766, 741 cm^−1^; ^1^H NMR (400 MHz, CDCl_3_): 7.55–6.87 (m, 3H, Ar), 2.75 (s, 2H, CH_2_), 2.09 (s, 3H, CH_3_); anal. calcd for C_9_H_8_ClNS: C 54.68, H 4.08, N 7.09, S 16.22; found: C 54.72, H 4.00, N 7.13, S 16.25.

*6-Chloro-3-phenyl-2H-1,4-benzothiazine* (**3m**). Mp: 64-65°C; IR (KBr) **ν**: 2932, 1649, 1477, 767, 738 cm^−1^; ^1^H NMR (400 MHz, CDCl_3_): 7.49–6.82 (m, 8H, Ar), 3.82 (s, 2H, CH_2_); anal. calcd for C_14_H_10_ClNS: C 64.73, H 3.88, N 5.39, S 12.34; found: C 64.82, H 3.79, N 5.42, S 12.38.

## Figures and Tables

**Table 1:** Catalyst optimization for the synthesis of 3-phenyl-2*H*-1,4-benzothiazine^a^.

Entry	Catalyst	Mg/mmol	Time/h	Yield/%^b^
1	—	—	24	42
2	Sulfonic acid functionalized nano-*γ*-Al_2_O_3_	25	4	81
3	Sulfonic acid functionalized nano-*γ*-Al_2_O_3_	50	3	96
4	Sulfonic acid functionalized nano-*γ*-Al_2_O_3_	100	3	96
5	Sulfonic acid functionalized nano-*γ*-Al_2_O_3_	150	3	95
6	Nano-*γ*-Al_2_O_3_	50	24	53
7	*p*-TsOH	50	5	79
8	Sulfamic acid	50	5	81
9	Silica sulfuric acid	50	6	82

^a^Reaction conditions: *o*-aminothiophenol (1 mmol), 2-bromo-1-phenyl-ethanone (1 mmol); EtOH (10 mL); reflux.

^b^Isolated yield.

**Table 2:** Solvent optimization for the synthesis of 3-phenyl-2*H*-1,4-benzothiazine^a^.

Entry	Solvent	Temperature/°C	Time/h	Yield/%^b^
1	H_2_O	Reflux	24	12
2	CH_3_CN	Reflux	3	97
3	CH_2_Cl_2_	Reflux	8	85
4	THF	Reflux	6	88
5	EtOH	25	10	69
6	EtOH	40	8	75
7	EtOH	60	5	89
8	EtOH	Reflux	3	96

^a^Reaction conditions: *o*-aminothiophenol (1 mmol), 2-bromo-1-phenyl-ethanone (1 mmol); sulfonic acid functionalized nano-*γ*-Al_2_O_3_ (50 mg); reflux.

^b^Isolated yield.

**Table 3:** Preparation of 3-substituted-2*H*-1,4-benzothiazines^a^.

Entry	R^1^	R^2^	Time/h	Product	Yield/%^b^
1	H	C_6_H_5_	3	**3a**	96
2	H	4-Me-C_6_H_4_	2	**3b**	97
3	H	4-MeO-C_6_H_4_	2	**3c**	95
4	H	4-F-C_6_H_4_	4	**3d**	93
5	H	4-Cl-C_6_H_4_	4	**3e**	94
6	H	4-Br-C_6_H_4_	4	**3f**	90
7	H	4-NO_2_-C_6_H_4_	6	**3g**	89
8	H	2-MeO-C_6_H_4_	3	**3h**	90
9	H	3-NO_2_-C_6_H_4_	6	**3i**	88
10	H	3,4,5-(MeO)_3_-C_6_H_2_	6	**3j**	86
11	H	2-Benzofuryl	5	**3k**	91
12	Cl	CH_3_	10	**3l**	78
13	Cl	C_6_H_5_	3	**3m**	93
14	Cl	4-Me-C_6_H_4_	3	**3n**	95
15	Cl	4-MeO-C_6_H_4_	3	**3o**	93
16	Cl	4-Cl-C_6_H_4_	4	**3p**	90
17	Cl	4-Br-C_6_H_4_	4	**3q**	87
18	Cl	2-Benzofuryl	4	**3r**	88
19	CF_3_	C_6_H_5_	4	**3s**	86
20	CF_3_	4-MeO-C_6_H_4_	4	**3t**	89

^a^Reaction conditions: *o*-aminothiophenol (1 mmol), *ω*-bromoketones (1 mmol); sulfonic acid functionalized nano-*γ*-Al_2_O_3_ (50 mg); EtOH (10 mL); reflux.

^b^Isolated yield.
